# Supplementary material for: 3D chromatin maps of a brown alga reveal U/V sex chromosome spatial organization
Source: Nat Commun. 2024 Nov 6;15:9590. doi: 10.1038/s41467-024-53453-5 (PMC11541908; doi:10.1038/s41467-024-53453-5)
Supplement: Supplementary file 1 — supplementary information [file 41467_2024_53453_MOESM1_ESM.pdf]

Supplementary Information

3D chromatin maps of a brown alga reveal U/V sex chromosome spatial organization

Liu et al.

Table of Contents

Supplementary Fig. 1. Schematic view of the approach used to reach a high-quality genome assembly of male and female *Ectocarpus*.

Supplementary Fig.2: Pedigree of the *Ectocarpus* strains used in this study. SP, diploid sporophyte; GA, gametophyte; m, male gametophyte; f, female gametophyte.

Supplementary Fig.3. Distribution of telomere repeat motif (TTAGGG or CCCTAA) in the *Ectocarpus*. haploid genome.

Supplementary Fig.4. The organization of *Ectocarpus* subtelomeres.

Supplementary Fig.5. Quality control of biological replicates of Hi-C data.

Supplementary Fig.6. No prominent TAD patterns are observed in *Ectocarpus*.

Supplementary Fig.7. Centromeres form distinct sub-compartments.

Supplementary Fig.8. Sequence alignment between the U and V sex chromosomes.

Supplementary Fig.9. Sequence characteristics of *Ectocarpus* centromeres.

Supplementary Fig.10. Heatmaps of histone marks around centromeres.

Supplementary Fig.11. Examples of virtual 4C-like plot of H3K79me2 domains larger than 100 kb.

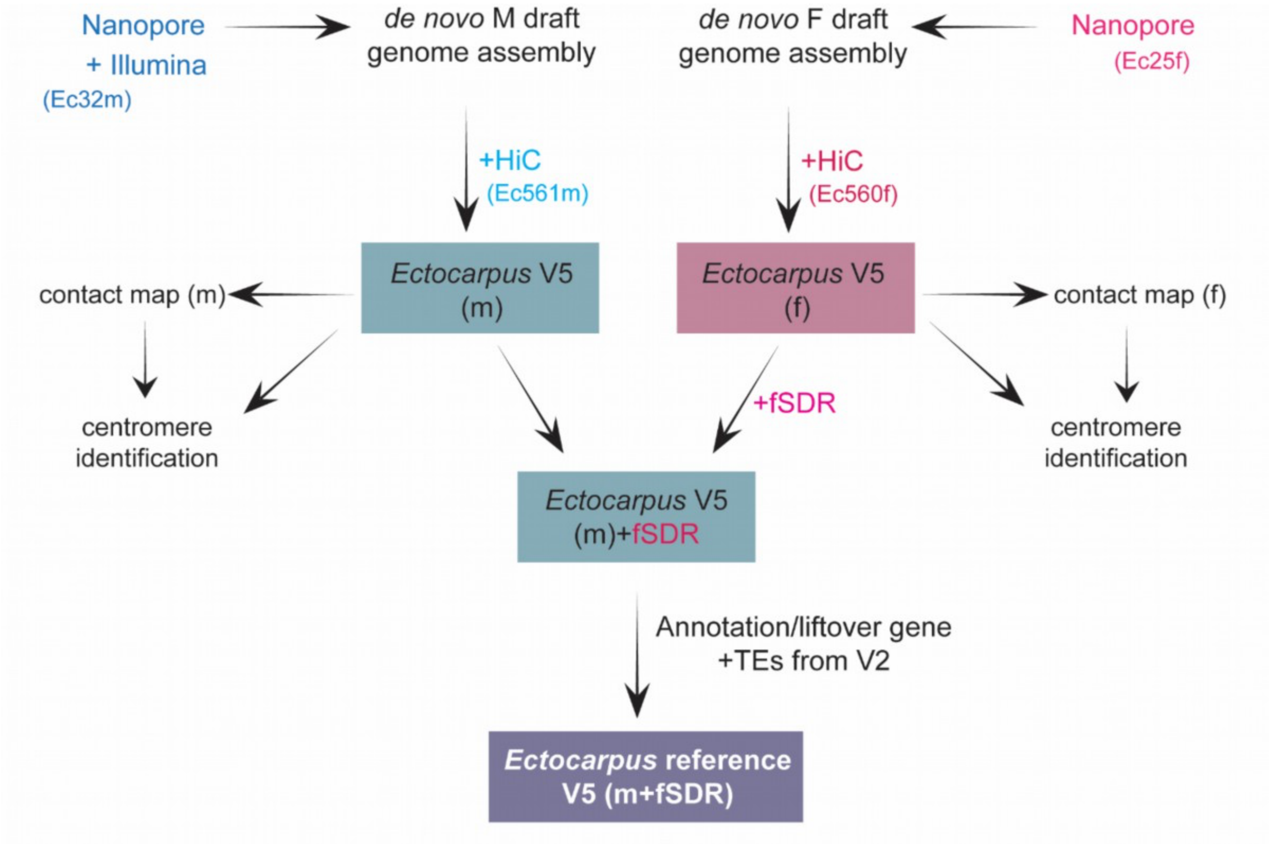

Supplementary Fig. 1: Schematic view of the approach used to reach a high-quality genome assembly of male and female *Ectocarpus*. De novo draft genomes from male and female siblings (Ec32 male and Ec25 female) were generated using Nanopore, and the male genome was polished using illumine reads. Genomes were further assembled using Hi-C data from Ec560 and Ec561 (near isogenic male and female lines[1]. This resulted in the generation of male and female V5 genomes that were used for producing high resolution male and female contact maps. In order to have only one 'reference' genome, we chose to use the male V5 reference genome and complemented it with the female-specific sex-determining contig (SDR). Therefore, the final 'reference' *Ectocarpus* genome V5 is composed of high-quality male genome that includes both the male and the female SDR. Annotation of this V5 reference genome was performed by lifting over gene and TEs annotations from the *Ectocarpus* V2 genome[2]. Centromeres were identified based on the contact maps and their annotation further refined (see methods for details). *Ectocarpus* strain numbers are given inside brackets (see also **Supplementary Table** 1).

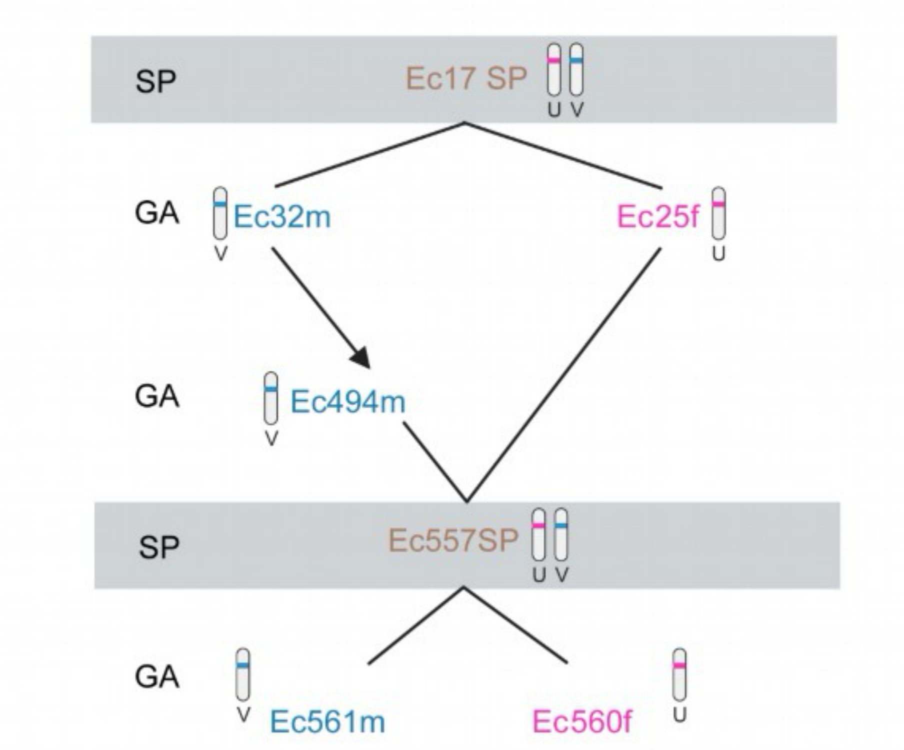

Supplementary Fig. 2: Pedigree of the *Ectocarpus* strains used in this study. *SP, diploid sporophyte; GA, gametophyte; m, male gametophyte; f, female gametophyte.*

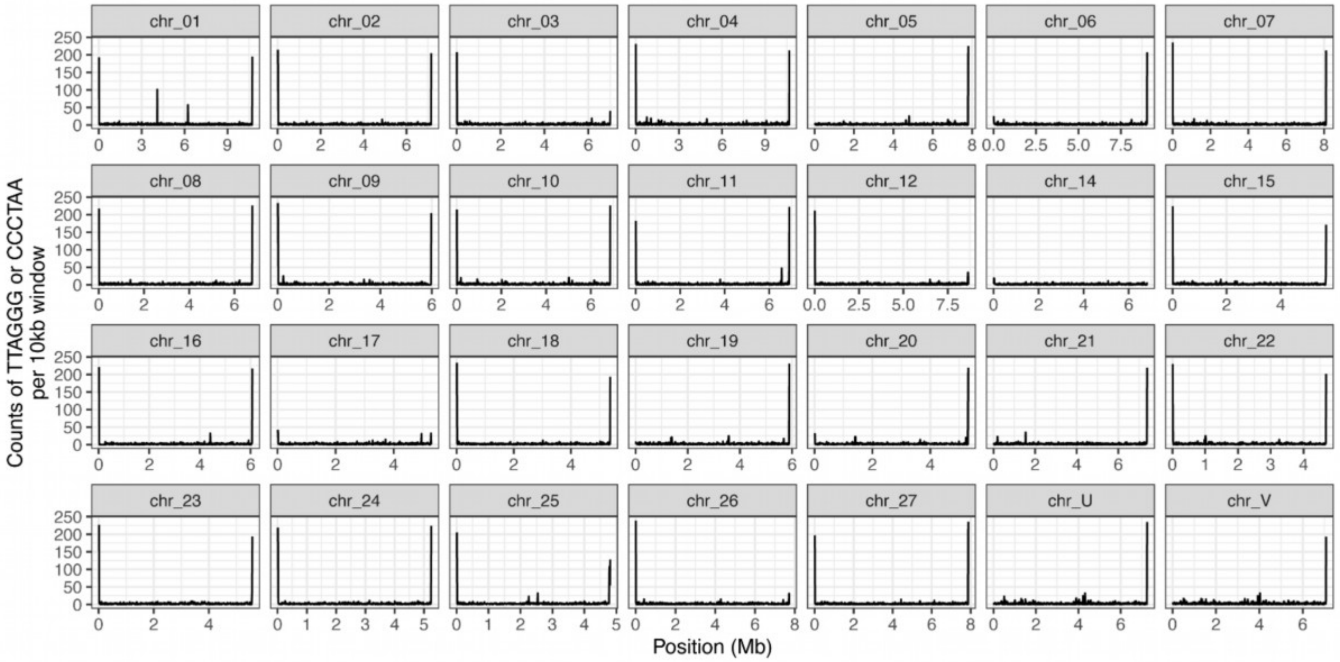

Supplementary Fig. 3: Distribution of telomere repeat motif (TTAGGG or CCCTAA) in the *Ectocarpus* haploid genome.

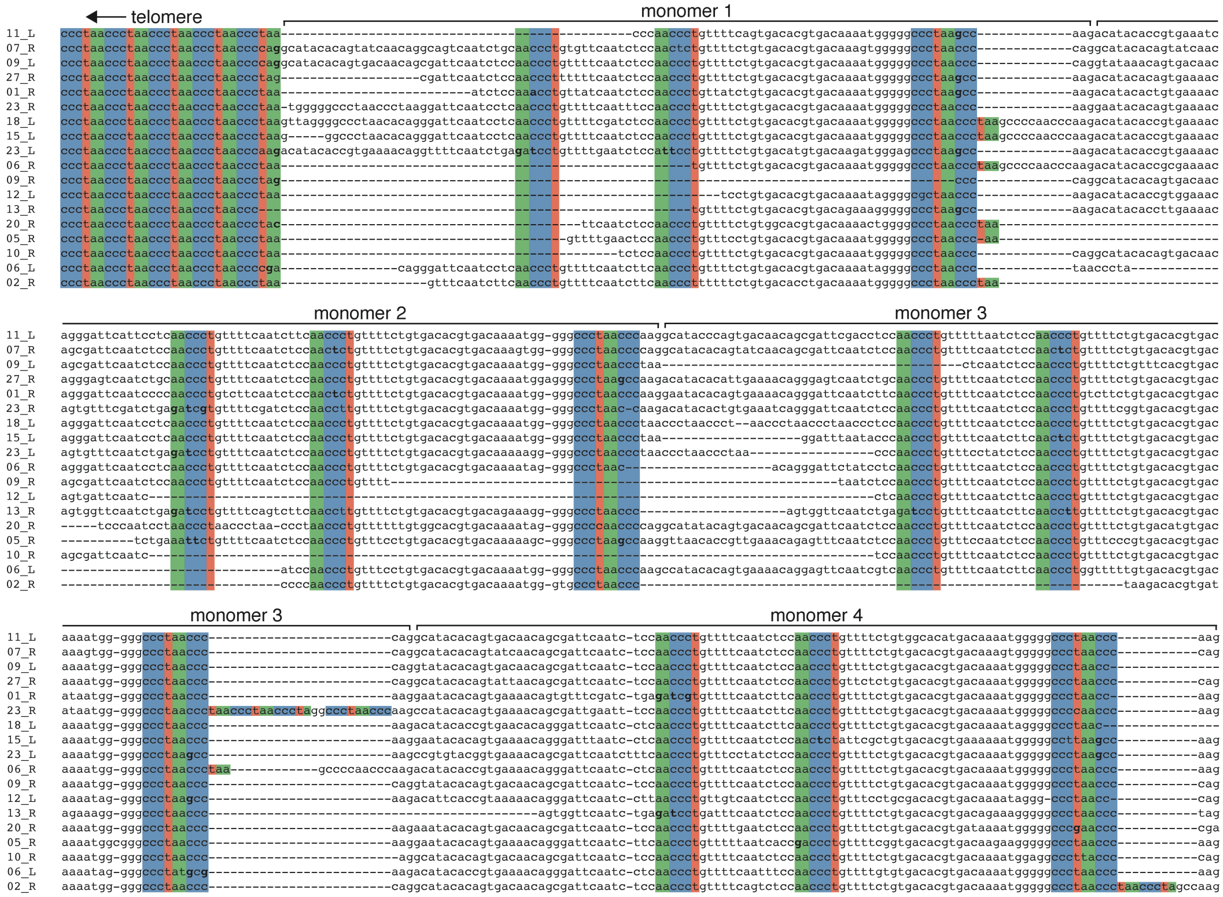

Supplementary Fig. 4: The organization of *Ectocarpus* subtelomeres. Alignment of 18 subtelomeres (e.g. 11_L is the left extremity of chromosome 11) showing the transition from the telomere to the subtelomeric satellite. The first four monomers of the ~98 bp satellite are shown. Telomeres and telomeric motifs present in the subtelomeric satellite are colored.

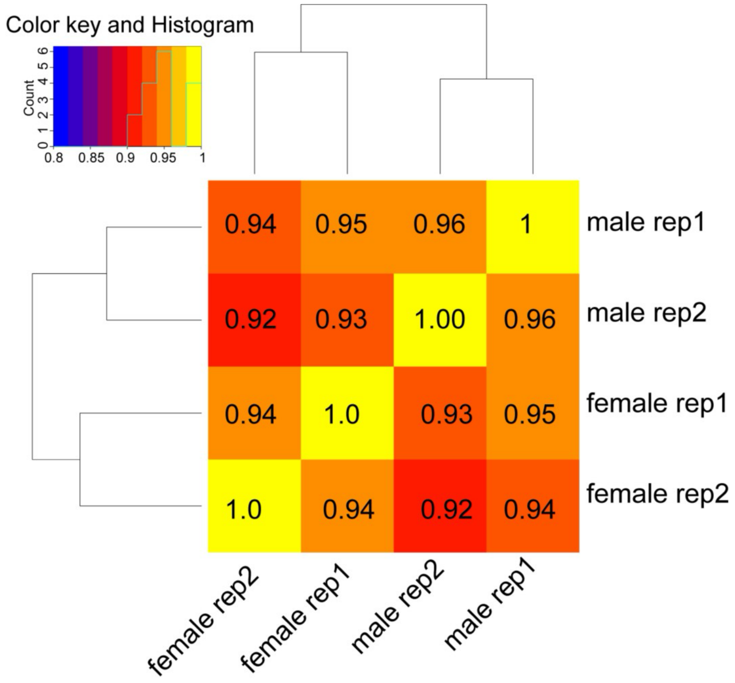

Supplementary Fig. 5: Quality control of biological replicates of Hi-C data. Pearson correction of biological replicates of *Ectocarpus* male and female Hi-C data at 10k bin size**.**

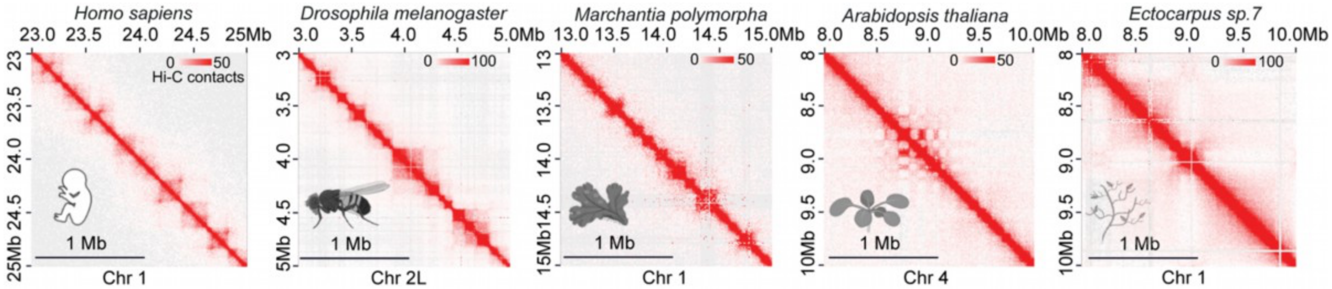

Supplementary Fig. 6: No prominent TAD patterns are observed in *Ectocarpus*. Examples of Hi-C maps from different species representing TADs patterns in *H. sapiens* (chromosome 1,) *Drosophila* (chromosome 2L,[3]), *Marchantia* (chromosome 1,[4]), *Arabidopsis* (chromosome 4,[5]) and *Ectocarpus* (chromosome 4, our paper). HiC maps of the different species were obtained using Juicerbox[6] at 10kb resolution. Note that TADs are not an obvious feature of *Arabidopsis* nor *Ectocarpus* genomes.

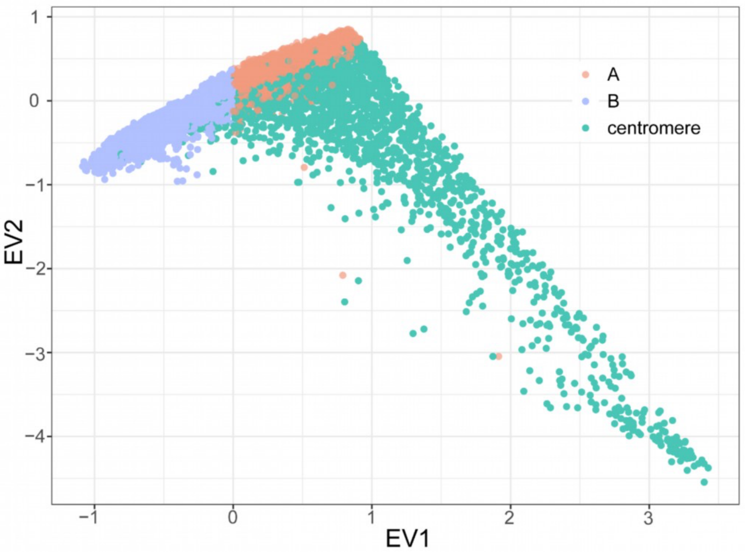

Supplementary Fig. 7: Centromeres form distinct sub-compartments. Centromere is represented as a sub-compartment, which could be separated from compartments by E1(PC1) and E2(PC2).

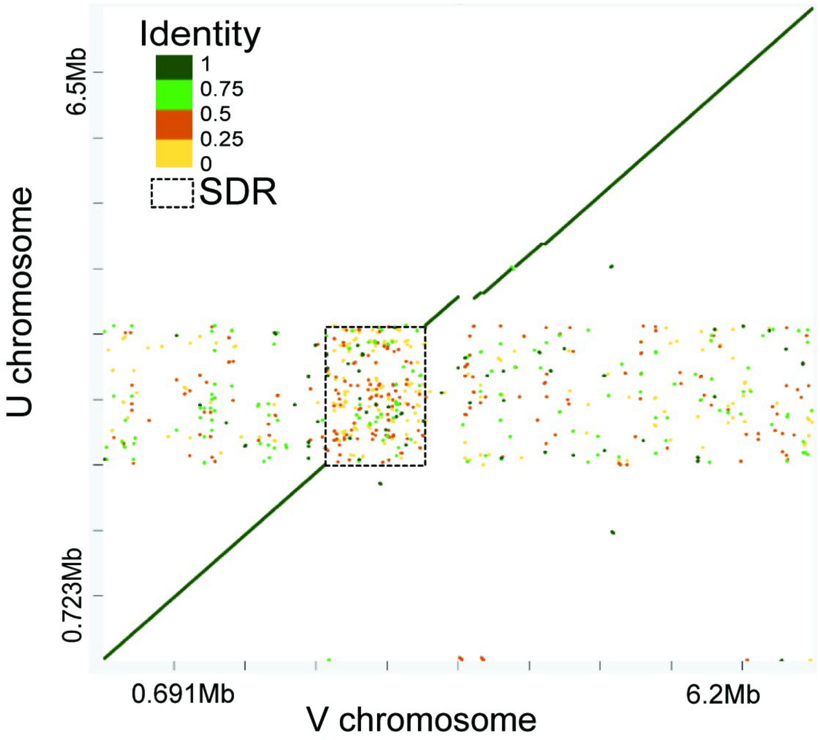

Supplementary Fig. 8: Sequence alignment between the U and V sex chromosomes. The matches are presented as colored lines. The colors correspond to identity values that have been clustered in four groups (below 25%, between 25% and 50%, between 50% and 75% and over 75%), the dash box shows SDRs.

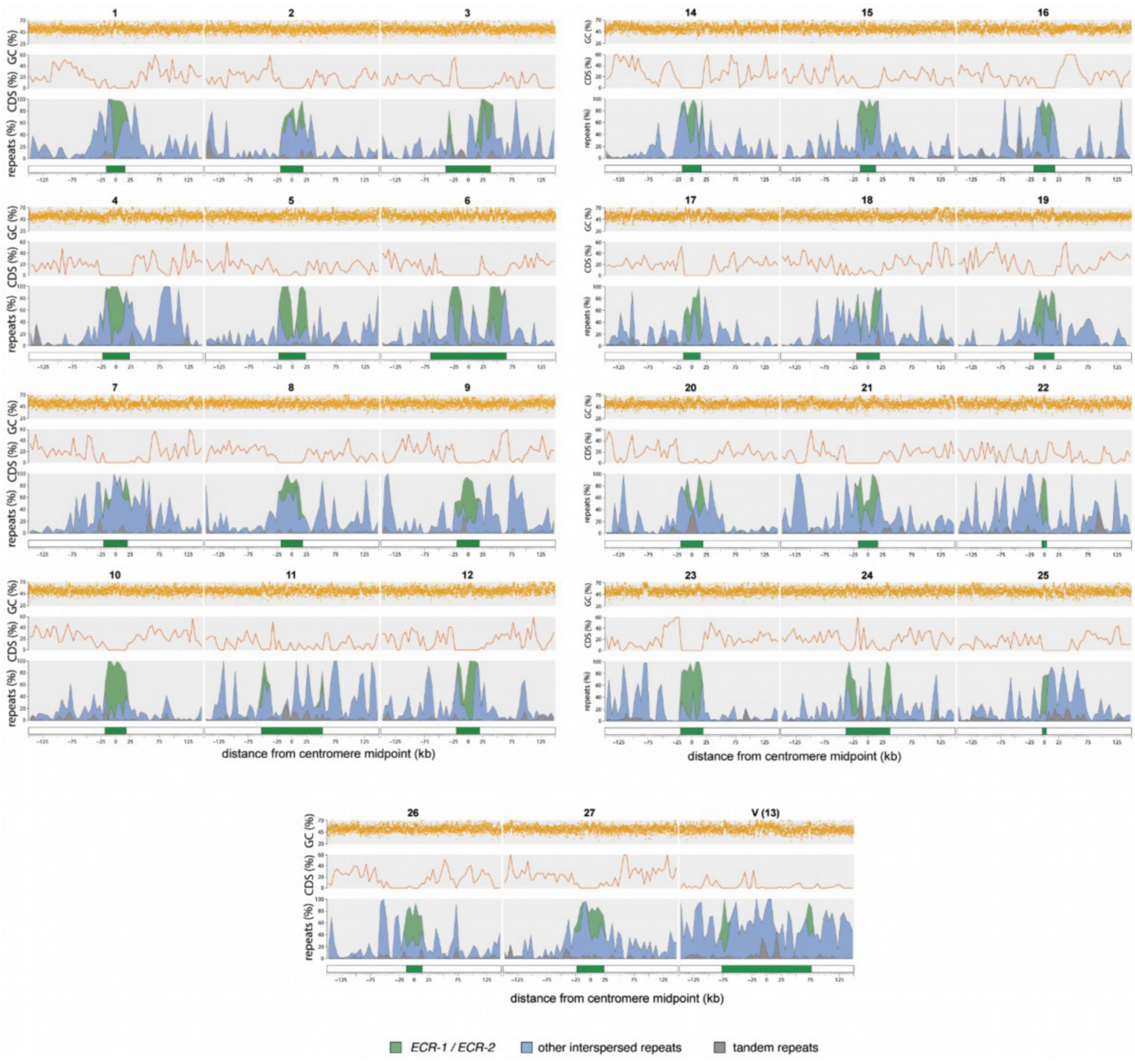

Supplementary Fig. 9: Sequence characteristics of *Ectocarpus* centromeres. Putative centromeres and flanking regions for all chromosomes from the male Ec32 V5 assembly. The centromere (green box) is defined as the region from the first to the last copy of *ECR* elements. The repeats panel is shown as a stacked area plot, and the percentage of each repeat type is plotted in 5 kb windows. Coding sequence (CDS) density is plotted in 5 kb windows, and GC content is plotted in 100 bp windows.

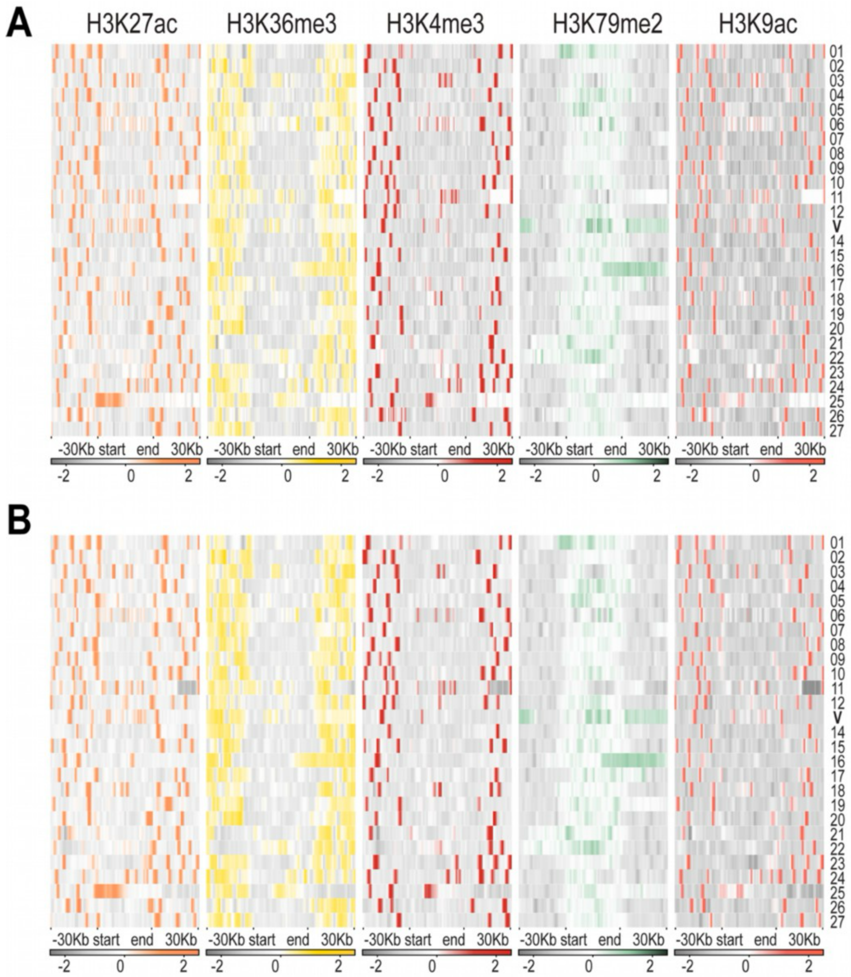

Supplementary Fig. 10: Heatmaps of histone marks around centromeres. For each heatmap, the log2(ChIP/input) is plotted over the putative centromeres and their 30Kb surrounding regions using a bin size of 100bp using uniquely mapped (A) and multi-mapped reads (B).

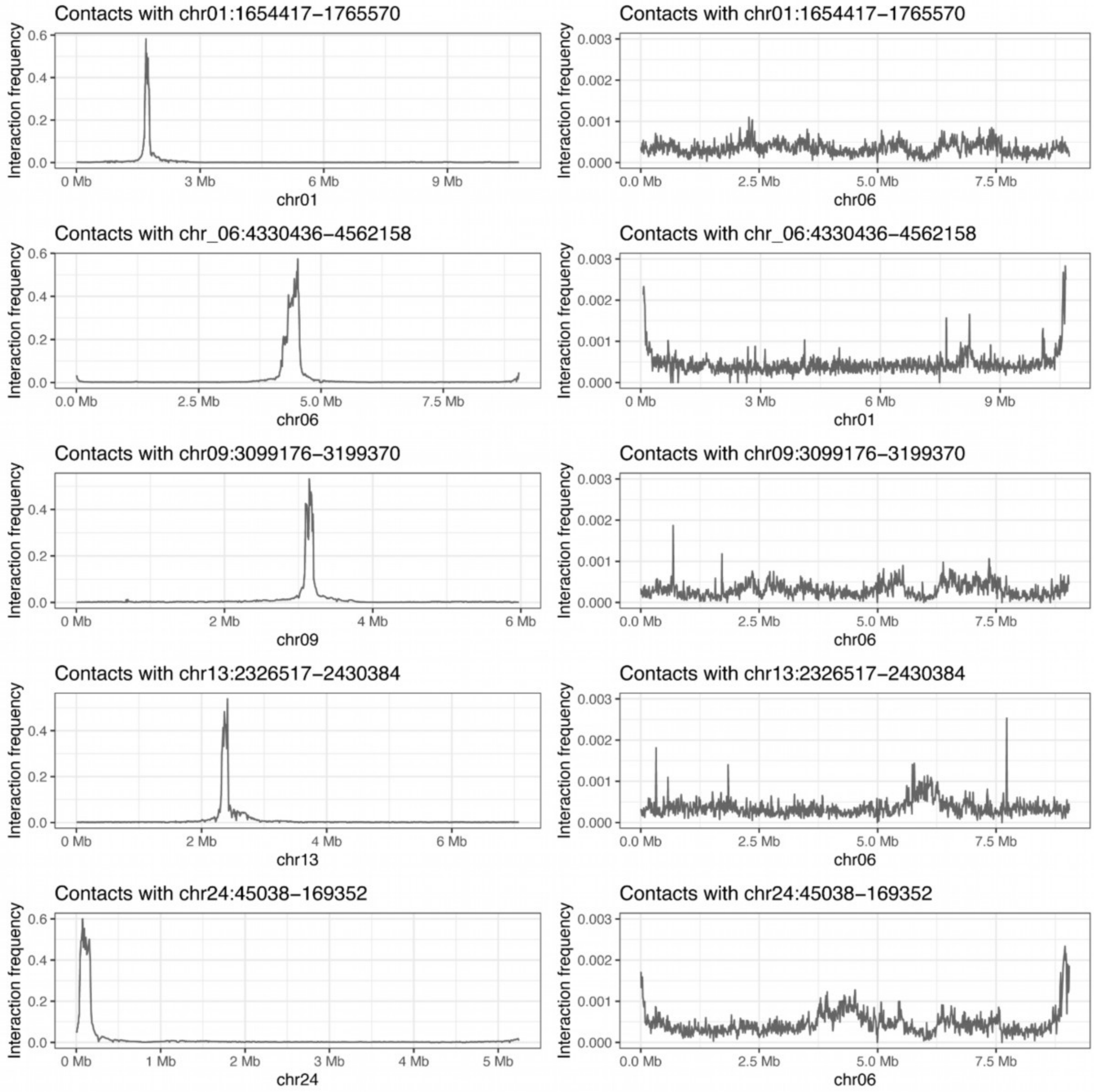

Supplementary Fig. 11: Examples of virtual 4C-like plot of H3K79me2 domains larger than 100 kb. Intra/inter chromosomal interaction frequency of H3Km79me2 domains.

Supplementary References

1. Gueno, J. *et al.* Chromatin landscape associated with sexual differentiation in a UV sex determination system. *Nucleic Acids Res* **50**, 3307–3322 (2022).
2. Cormier, A. *et al.* Re-annotation, improved large-scale assembly and establishment of a catalogue of noncoding loci for the genome of the model brown alga Ectocarpus. *New Phytologist* **214**, (2017).
3. Ray, J. *et al.* Chromatin conformation remains stable upon extensive transcriptional changes driven by heat shock. *Proc Natl Acad Sci U S A* **116**, (2019).
4. Karaaslan, E. S. *et al.* Marchantia TCP transcription factor activity correlates with three-dimensional chromatin structure. *Nat Plants* **6**, 1250–1261 (2020).
5. Yin, X. *et al.* Binding by the Polycomb complex component BMI1 and H2A monoubiquitination shape local and long-range interactions in the Arabidopsis genome. *Plant Cell* (2023) doi:10.1093/plcell/koad112.
6. Durand, N. C. *et al.* Juicebox Provides a Visualization System for Hi-C Contact Maps with Unlimited Zoom. *Cell Syst* **3**, 99–101 (2016).
