# Supplementary material for: 3D chromatin maps of a brown alga reveal U/V sex chromosome spatial organization
Source: Nat Commun. 2024 Nov 6;15:9590. doi: 10.1038/s41467-024-53453-5 (PMC11541908; doi:10.1038/s41467-024-53453-5)
Supplement: Supplementary file 4 — Description of Additional Supplementary Files [file 41467_2024_53453_MOESM4_ESM.pdf]

Description of Additional Supplementary Files:

Supplementary Data 1: Ectocarpus strains used in this study.

Supplementary Data 2: Nanopore long reads statistics.

Supplementary Data 3: Statistics of number of Hi-C reads in the different samples and replicates

Supplementary Data 4: Genome statistics (chromosome level assembly).

Supplementary Data 5: Statistics of complete, single, duplicated, fragmented, and missing genes computed by BUSCO v5.7.0

Supplementary Data 6: Genomic coordinates and repeat densities for Ectocarpus putative centromeres. Centromeric coordinates were defined as the first to the last copy of ECR elements.

Supplementary Data 7: Gene expression levels (transcripts per million, TPM) in the Ectocarpus male versus female samples used in this study (gametophytes)

Supplementary Data 8: Number of sex biased genes (SBG) in compartments A and B in males and females.

Supplementary Data 9: Comparative statistics of Ectocarpus sex determination region (SDR) annotations between the Ectocarpus V2 and V5 assembly.
